# Common agricultural weeds among alien invasive plants in China: Species lists and their practical managing strategies

**DOI:** 10.1016/j.heliyon.2025.e41772

**Published:** 2025-01-07

**Authors:** Zeyue Huang, Min Lin, Guoqi Chen

**Affiliations:** a Jiangsu Key Laboratory of Crop Genetics and Physiology/Jiangsu Key Laboratory of Crop Cultivation and Physiology, Agricultural College (Research Institute of Rice Industrial Engineering Technology) of Yangzhou University, Jiangsu Co-Innovation Center for Modern Production Technology of Grain Crops, Yangzhou University, Yangzhou, China; b Nanjing Kelihua Middle School Tangcheng Branch, Nanjing, China

**Keywords:** Alien invasive plant, Herbicide, Highland, Non-chemical control, Paddy field, Plantation

## Abstract

Plant invasion is a big challenge to weed management of agricultural lands. In order to reveal the list of common weed species among alien invasive plants, and reveal practical management strategies, we extracted the species lists of common alien agricultural weeds (CAAWs) of various arable lands and plantations, by comparing the lists of alien invasive plant species and common weed species published in China. Totally 88 species from 18 families were recognized as CAAWs, among which 43.0 % are native to North America, followed by South America (34.4 %), Europe (29.0 %), Asia (23.7 %) and Africa (17.2 %); 62.4 % were introduced into China from 1840 to 1949. CAAWs such as *Aegilops tauschii* Coss., *Alopecurus myosuroides* Huds., *Lolium multiflorum* Lamk, *Avena fatua* L.*, Phalaris minor* Retz, *Phalaris paradoxa* L., *Veronica persica* Poir.*, Geranium carolinianum* L.*, Ranunculus muricatus* L.*,* and *Cerastium glomeratum* Thuill. frequently infest highlands with summer-ripe crops such as wheat and oilseed rape; *Alternanthera* spp.*, Panicum repens* L., *Paspalum conjugatum* Bergius, and *Ageratum conyzoides* L. frequently infest highlands with autumn-ripe crops; and *Paspalum distichum* L., *Alternanthera philoxeroides*, and *Ammannia coccinea* Rott. occasionally infest rice fields. Troublesome CAAWs in plantations in China mainly consist of tall herbs, and climbing or spiny plants, such as *Mikania micrantha* Kunth*, Ipomoea* spp.*, Solidago canadensis* L.*, Erigeron* spp*.* and *Bidens* spp. Management strategies against CAAWs in current China mainly rely on chemical control, tillage, soil mulching, and manual removing of weeds. Next, effective risk assessing models targeting to different sorts of arable lands or plantations are urgently needed; as well, effective, feasible and sustainable integrated management strategies against troublesome CAAWs should be developed and applied.

## 1 Introduction

Plant invasion has been one of the biggest ecological challenges in China [1]. The checklist of alien invasive plant species was reported to be 108 in 2000 [2], increased to 270 in 2008 [3], and updated to 403 in 2023 [1]. Among the 403 alien invasive plant species, 48 were introduced into China after 2000 [1]. Meanwhile, the diffusion of invasive plants has been accelerating with the increasing construction of traffic systems, increasing cross-region flows of people, transport vehicles, and goods, and the introduction of new alien plant species [4]. Many invasive plants have infested agricultural lands and evolved herbicide resistance, which has caused severe challenges to local agriculture [[5], [6], [7]].

Weeds are one of the most significant biological threats to agriculture; Oerke reported that they cause approximately 34 % of crop yield losses worldwide [8]. Moreover, weed management is one of the most labor-intensive and costly operations in crop production. With the decreasing availability of labor for agricultural work in China, weed management is becoming more difficult and expensive [7]. Agriculture in China has been shifting from traditional small farms devoted a great manpower to more giant farms with agricultural mechanization [9]. Thus, weed communities in agricultural lands also changed accordingly [10]. Many common or problematic weed species are effectively managed through updated weed management strategies that focus on chemical control. As a result, a range of ecological niches in agricultural lands is released. Weeds adapted to chemical control strategies have become increasingly troublesome [5,7]. Some alien invasive plant species infesting agricultural areas might become serious agricultural weeds in China, such as *Lolium multiflorum* Lamk. [5], *Alopecurus myosuroides* Huds. [5], and *Mikania micrantha* Kunth [11,12]. Alien plant invasions further increase the difficulty and complexity of weed management in agricultural lands. Qiang and Zhang reviewed species of invasive plants in agroecosystems in China and their management strategies, which included 331 plant species and five varieties [13]. Different alien invasive plant species showed great differences in harmfulness and practical managing strategies. Managing strategies against alien invasive plants in agricultural areas are mainly agronomic and chemical measures. Bio-herbicides against agricultural weeds have not been used in large areas, due the low efficacy and high costs.

Considering limitations in managing resources, knowledge of the list of common weed species among alien invasive plants and their practical managing strategies is important for focusing limited resources to manage troublesome alien agricultural weeds. In China, many monographs and publications reported common weed species and their managing strategies in agricultural areas, among which “The lists of agricultural weeds are cited from the Encyclopedia of Plant Protection in China - Weed Volume” [7], “Weed Flora of China” [6], and “Crop diseases and insect pests in China” [14]are the most important ones. Also, many publications reported the species lists of alien invasive plants, among which “Invasive Alien Plants in China: an update” [1], and “Alien Invasive Flora of China” [15] are the newest updates. Moreover, all chemical herbicides registered and their applying methods in China could be searched in the database of China Pesticide Information Network (www.chinapesticide.org.cn/zwb/dataCenter). Therefore, we extracted the species lists of common alien agricultural weeds (CAAWs) of various arable lands and plantations, by comparing the lists of alien invasive plant species and common weed species; as well, we searched references on the managing strategies and herbicide resistance of each species of CAAW in database of “Web of Science” (webofscience.clarivate.cn) and “CNKI” (www.cnki.net/). The objectives of this study were to: 1) reveal CAAW species list in China, which have been troublesome in certain agricultural areas in China; and 2) reveal relevant management methods against CAAWs that are currently effective, feasible, and publicly available.

## 2 Materials and methods

The list of alien invasive plant species is mainly cited from “Invasive Alien Plants in China: an update” [1] and “Alien Invasive Flora of China” [15]. The lists of agricultural weeds are cited from the Encyclopedia of Plant Protection in China - Weed Volume [7], Weed Flora of China [6], and Crop diseases and insect pests in China [14]. We got the list of common CAAWs by comparing the lists of alien invasive plant species with common weed species in China from the above references (Table 1). Moreover, in 2020, we observed *Geranium dissectum* L. infesting an oilseed rape land, which is a newly recorded alien invasive plant species [16]. Thus, we added *Geranium dissectum* to the list of CAAW species in China. According to “Alien Invasive Flora of China” [15], *Vicia sativa* L., *Sonchus oleraceus* L., *Medicago minima* (L.) Lam.*, Celosia argentea* L., and *Veronica peregrina* L. are native to China, and *Coriandrum sativum* L. is also excluded from the list, for being a common vegetable plant species with a shallow threat to agriculture. For each CAAW species included, plant families were determined according to Flora of China [17]; infesting habitats were determined according to relative references mentioned above [6,7,14]; and native ranges, entry periods, and modes of reproduction were determined according to “Alien Invasive Flora of China” [15] and “Flora of China” [17].

**Table 1:** Alien invasive weed species in paddy fields (PD), dry crop lands (DL) and plantations (PL) in China.

Family	Species	Infesting habitat^a^	Native range^b^	Entry period^c^	Reproduction mode^d^
Amaranthaceae	*Alternanthera philoxeroides*	PF, DL, PL	SA	M	V
	*Amaranthus ascendens*	DL, PL	Af, As, Eu	A	S
	*Amaranthus retroflexus*	DL, PL	NA	M	S
	*Amaranthus spinosus*	DL	NA, SA	A	S
	*Amaranthus viridis*	DL, PL	NA, SA	M	S
	*Gomphrena celosioides*	DL	NA, SA	M	S, V
	*Amaranthus hybridus*	DL	NA, SA	M	S
Apiaceae	*Daucus carota*	PL	Eu	A	S
Asteraceae	*Ageratum conyzoides*	DL, PL	SA	M	S
	*Ageratum houstonianum*	DL	NA	M	S
	*Bidens alba*	DL, PL	NA, SA	M	S
	*Bidens bipinnata*	PL	NA, SA	M	S
	*Bidens frondosa*	DL, PF	NA	M	S
	*Bidens pilosa*	PL	As, NA, SA	M	S
	*Bidens tripartita*	PF	As, Eu	A	S
	*Chenopodium glaucum*	DL	Eu	M	S
	*Chromolaena odorata*	PL	NA	M	S
	*Cichorium intybus*	DL	Af, As, Eu	M	S
	*Erigeron annus*	PL	NA	M	S
	*Erigeron bonarinsis*	PL	SA	M	S
	*Erigeron canadensis*	PL	NA	M	S
	*Erigeron philadelphicus*	PL	NA	C	S
	*Erigeron sumatrensis*	PL	SA	C	S
	*Eupatorium catarium*	DL, PL	SA	C	S
	*Flaveria bidentis*	DL	SA	C	S
	*Galinsoga parviflora*	DL, PL	SA	M	S
	*Galinsoga quadriradiata*	DL, PL	NA	M	S
	*Gynura crepidioides*	PL	Af	M	S
	*Mikania micrantha*	PL	NA, SA	M	S
	*Parthenium hysterophorus*	DL, PL	NA, SA	M	S
	*Senecio vulgaris*	DL	Eu	M	S
	*Solidago canadensis*	DL, PL	NA	M	S, V
	*Soliva anthemifolia*	DL	SA	M	S
	*Sonchus asper*	DL, PL	Af, As, Eu	M	S
	*Synedrella nodiflora*	DL	NA, SA	M	S
	*Wedelia trilobata*	PL	SA	C	V
	*Xanthium chinense*	DL, PL	NA	M	S
Brassicaceae	*Coronopus didymus*	DL	SA	M	S
	*Lepidium virginicum*	DL	NA	M	S
**Convolvulaceae**	*Cuscuta campestris*	DL	NA	C	S
	*Cuscuta epilinum*	DL	Eu, NA	M	S
	*Ipomoea cairica*	PL	Af, As	M	S, V
	*Ipomoea triloba*	DL, PL	NA, SA	M	S
	*Ipomoea nil*	DL, PL	NA, SA	A	S
	*Ipomoea purpurea*	DL, PL	NA, SA	M	S
Caryophyllaceae	*Agrostemma githago*	DL	Af, As, Eu	M	S
	*Cerastium glomeratum*	DL	Eu	A	S
	*Vaccaria segetalis*	DL	Eu	A	S
Cucurbitaceae	*Sicyos angulatus*	DL	NA	C	S
Euphorbiaceae	*Euphorbia hirta*	DL	NA, SA	A	S
	*Euphorbia supina*	DL, PL	NA	M	S
Fabaceae	*Mimosa pudica*	DL	NA, SA	A	S
	*Medicago hispida*	DL	Af, As, Eu	M	S
	*Medicago sativa*	DL	As	A	S
	*Melilotus officinalis*	DL	As, Eu	M	S
	*Trifolium repens*	PL	Af, As, Eu	M	S, V
Geraniaceae	*Geranium carolinianum*	DL, PL	NA	M	S
	*Geranium dissectum*	DL	Eu	C	S
Lythraceae	*Ammannia coccinea*	PF	NA	C	S
Malvaceae	*Abutilon theophrasti*	DL, PL	As	A	S
	*Corchorus olitorius*	DL, PL	Af	M	S
	*Hibiscus trionum*	DL	Af	A	S
	*Sida spinosa*	PL	NA	C	S
Phytolaccaceae	*Phytolacca americana*	PL	NA	M	S, V
Plantaginaceae	*Plantago virginica*	PL	NA	M	S
	*Plantago lanceolata*	PL	As,Eu	M	S
	*Veronica arvensis*	DL	Eu	M	S
	*Veronica persica*	DL, PL	Eu	M	S
	*Veronica polita*	DL	As	A	S
Poaceae	*Paspalum distichum*	PF	NA, SA	M	S, V
	*Aegilops tauschii*	DL	As, Eu	C	S
	*Alopecurus myosuroides*	DL	As, Eu	C	S
	*Avena fatua*	DL	As, Eu	M	S
	*Chloris virgata*	DL	NA,SA	M	S
	*Lolium multiflorum*	DL	Af, As, Eu	M	S
	*Lolium temulentum* var. *longiaristatum*	DL	Af, As, Eu	M	S
	*Panicum dichotomiflorum*	DL, PF	NA	M	S
	*Panicum repens*	DL	Af, Eu	A	V
	*Paspalum conjugatum*	DL	Af	M	S, V
	*Paspalum dilatatum*	PL	SA	M	S
	*Phalaris minor*	DL	Af, As, Eu	C	S
	*Phalaris paradoxa*	DL	Af, As, Eu	C	S
Ranunculaceae	*Ranunculus muricatus*	DL	As, Eu	M	S
Rubiaceae	*Richardia scabra*	PL	NA, SA	C	S
	*Spermacoce alata*	PL	SA	M	S, V
Solanaceae	*Physalis angulata*	DL, PL	SA	A	S
	*Physalis peruviana*	DL, PL	SA	M	S
	*Solanum rostratum*	DL	NA	C	S

a PF = paddy field, DL = dry land, and PL = plantation.

b Af = Africa, As = Asia, Eu = Europe, NA = North America, and SA= South America.

c A = Ancient (before 1840), Modern (1840–1949), and C = contemporary (after 1949).

d S = seed, and V = vegetative reproduction.

## 3 Results and discussion

### 3.1 Species list of common alien agricultural weeds (CAAWs) in China

Eighty-eight species were identified as CAAW (Common Agricultural Alien Weeds) species. The 88 CAAWs do not include all alien invasive plant species on agricultural lands. At the same time, all of them are common weed species in agricultural lands in some areas of China that need to be controlled in many situations.

**Fig. 1:**
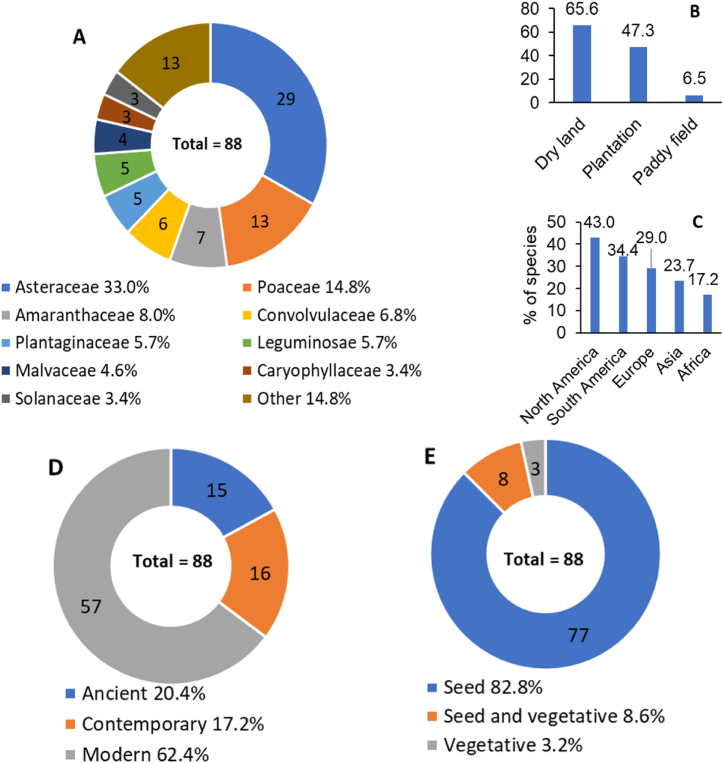
Characteristics of exotic agricultural weed species in China. A. family composition, B: infesting habitat, C: native range, D: entry time (Ancient = before 1840, modern = 1840–1949, contemporary = after 1949), E: main reproduction mode on field.

The 88 CAAW species are from 18 families, among which 33.0 % are from Asteraceae, and 14.8 % from Poaceae (Fig. 1A), 65.6 % are common in dry lands, 47.3 % are standard in plantations, and 6.5 % are expected in paddy fields (Fig. 1B). As to native ranges (Figs. 1C), 43.0 % are native to North America, followed by South America (34.4 %), Europe (29.0 %), Asia (23.7 %) and Africa (17.2 %); 62.4 % were introduced into China from 1840 to 1949 (Fig. 1D), followed by earlier than 1840 (20.4 %) and after 1949 (17.2 %). About 83 % of CAAW species reproduce mainly by seeds (Fig. 1E).

The list of CAAW species showed higher representations of Asteraceae, Poaceae, Amaranthaceae, and Plantaginaceae compared with the list of alien invasive plant species [1]. Considering many crop species are from Poaceae and Asteraceae, many of the world's worst weed species are from these two families [18]. Amaranthaceae is a family that holds many of the world's worst weed species [18], and many severe agricultural weed species of this family are not native to China [1]. The order of species richness referring to native ranges of CAAWs was the same as those of alien invasive plant species [1]. In contrast, a higher percentage of CAAW species were native to Eurasia. Regarding introducing periods, 62.4 % of CAAW species were introduced into China after 1949, and the percentage among the 403 alien invasive plant species was 41.4 % [1].

Alien plant invasions are widely regarded as a product of artificial activities [4]. The intensive artificial disturbance greatly facilitates establishing and distributing alien plants in agricultural lands. For instance, the moist and fertile soil usually maintained by growers provides favorable conditions for invasions of CAAWs. The cultivation of crops, particularly cereal crops such as rice (*Oryza sativa* L.), wheat (*Triticum aestivum* L.), corn (*Zea mays* L.), and oilseed crops like canola and soybean (*Glycine max* L.), has shifted towards mechanized and large-scale farming practices [19]. The invasions and infestation of CAAW species highly adapted to chemical control form one of the biggest challenges in modern cereal and oil crop cultivations. Firstly, weed species, including CAAWs that belong to the same plant family as the crops in the fields, tend to be more challenging to control chemically due to their similar sensitivity to various herbicides; fortunately many selective herbicides can effectively control weeds while being relatively safe for crops [20]. For example, in highland areas, wheat and corn belong to the Poaceae family, making CAAWs from this family particularly difficult to control. Similarly, oilseed rape and soybean are broadleaf crops, and broadleaf CAAWs also present significant challenges for chemical control. Secondly, CAAW species that frequently evolve herbicide resistance are often troublesome weeds. For example, *Geranium carolinianum* L. [21] and *Veronica persica* Poir. [22] are serious weed species in croplands cultivating summer-ripe broadleaf crops [11]. Similarly, several grassy CAAWs such as *Aegilops tauschii* Coss*.* [23,24], *Alopecurus myosuroides* [25,26] and *Lolium multiflorum* [27] are severe weeds for wheat lands. On the other hand, cultivating and management practices on agricultural lands are a barrier against plant invasions.

### 3.2 Species and practical management strategies of CAAWs in highlands

Sixty-one alien invasive plants are CAAW species in highlands (Table 1). Species composition within a community is determined by a series of ecological filters that sort species based on their traits. Highland crop species are very diverse, such as wheat, corn, soybean, oilseed rape (*Brassica rapa* L.), potato (*Solanum tuberosum* L.), various vegetables, cotton (*Gossypium hirsutum* L.), watermelon (*Citrullus lanatus* (Thunb.) Matsum. & Nakai), peanut (*Arachis hypogaea* L.), millet (*Setaria italica* var*. germanica* (Mill.) Schred.), sunflower (*Helianthus annuus* L.), and sorghum (*Sorghum bicolor* (L.) Moench), and thus weed communities are also diverse in relation with different crops and cultivating systems [7]. Different highland habitats host different weed communities [7] and might be infested by different CAAWs. Highland crops are usually annual or biennial, and periodic cultivating practices and weed management form barriers against CAAW invasions, such as tillage, crop harvesting, and rotation [7].

#### 3.2.1 Species of CAAW in highlands with summer-ripe crops

Summer-ripe crops are usually planted in autumn or winter and harvested in later spring or early summer [7]. Under integrated weed management relying on chemical control, weed communities in cereal croplands and broadleaf croplands differ significantly [7]. Wheat is the most critical summer-ripe cereal crop. Several CAAW species from the Poaceae family frequently cause severe yield losses of wheat in China (Table 1), including *Aegilops tauschii* [23,24], *Alopecurus myosuroides* [25,26], *Lolium multiflorum* [27], *Avena fatua* L. [28,29]*, Phalaris minor* Retz. and *Phalaris paradoxa* L. [30,31]. For example, *Aegilops tauschii*, similar to the genetic background of wheat, has strong reproductive ability and is easy to spread. It competes with wheat for sunlight, water, and fertilizer in wheat fields [32]. Zhang et al. reported that *Avena fatua* led to a 50–60 % decrease in wheat yield in Qinghai Province, China [33]. Liu et al. found that *Lolium multiflorum* caused yield losses of wheat ranging from 30 % to 90 % [34]. Fang et al. reported that when the density of *Alopecurus myosuroides* was 420 plants/m^2^, the spike density of wheat decreased by 55.2 % [35]. Some broadleaf CAAW species may occasionally infest wheat lands, such as *Veronica* spp. [36,37]and *Geranium carolinianum* [38,39]. Moreover, some CAAWs could also be common weed species in wheat lands in China, such as *Veronica arvensis* L.*, Sonchus asper* (L.) Hill*, Ranunculus muricatus* L.*, Cerastium glomeratum* Thuill.*,* and *Vaccaria segetalis* Sm. [6]. Oilseed rape is one of the most essential summer-ripe broadleaf crops. Several species of broadleaf CAAWs frequently cause severe yield losses, such as *Veronica persica, Geranium carolinianum, Ranunculus muricatus,* and *Cerastium glomeratum* [7,10]. Moreover, some CAAWs could also be common weed species in oilseed rape lands in China, such as *Veronica polita* Fries*, Veronica arvensis, Sonchus asper, Vaccaria segetalis*, *Lolium multiflorum, Avena fatua*, and *Geranium dissectum* [7,10].

#### 3.2.2 Species of CAAW in highlands with autumn-ripe crops

Autumn-ripe crops are planted in late spring or early summer and harvested in autumn, including cereal crops such as corn, millet, and sorghum and broadleaf crops such as soybean, cotton (*Gossypium hirsutum* L.), peanut, sunflower, watermelon, and most on-field vegetable species. Some CAAW species may occasionally be troublesome, such as *Alternanthera philoxeroides* (Mart.) Griseb. [40]*, Amaranthus retroflexus* L*.* [41]*, Amaranthus viridis* L.*, Amaranthus hybridus* L.*, Ageratum conyzoides* L*.* [[42], [43], [44]]*, Abutilon theophrasti* Medikus [45,46]*, Ipomoea triloba* L. [47]*, Galinsoga quadriradiata* Ruiz & Pavon*, Galinsoga parviflora* Cav. [48] *Panicum repens* L., *Paspalum conjugatum* Bergius, *Eupatorium catarium* (Hieron. ex Kuntze) R.M.King & H.Rob.*, Physalis angulate* L. [49]*, Physalis peruviana* L.*,* and *Ipomoea purpurea* (L.) Roth. For example, Francischini et al. found that *Amaranthus retroflexus* caused 5–34 % yield losses in corn and up to 46 % in sorghum [50]. Occurrences of broadleaf CAAWs in autumn-ripe cereal croplands are usually lower, for the high effectiveness of chemical control. Troublesome grassy weeds in autumn-ripe cereal croplands are mainly native species [7].

For autumn-ripe broadleaf croplands, many broadleaf CAAW species are troublesome, such as *Alternanthera philoxeroides* [40], *Amaranthus retroflexus* [51], *Amaranthus viridis, Amaranthus hybridus* [52]*, Amaranthus blitum* L.*, Abutilon theophrasti, Ageratum conyzoides* L. [53]*, Ipomoea nil* (L.) Roth [54]*, Ipomoea purpurea* [55]*,* and *Ipomoea triloba* [56,57]. For example, Aguyoh and Masiunas found that *Amaranthus retroflexus* caused a loss of up to 58 % at eight plants m^−2^ in snap bean (*Phaseolus vulgaris* L.) [58]. *Ipomoea nil* was reported to be a troublesome weed in soybean fields in Liaoning Province, China [59].

Some CAAW species are occasionally dominant in autumn-ripe broadleaf croplands, such as *Euphorbia supina* L., *Xanthium chinense* Mill., *Abutilon theophrasti, Physalis angulate*, and *Physalis peruviana* L. [6,7]. For highlands with greenhouse crops, *Alternanthera philoxeroides, Amaranthus ascendens, Veronica persica, Veronica polita,* and *Geranium carolinianum* are the most troublesome CAAWs [6,7]. Various CAAW species may be found in a greenhouse at low densities, such as *Erigeron* spp., *Sonchus asper*, *Bidens* spp., and *Veronica arvensis* [6].

#### 3.2.3 Management strategies against CAAWs in highlands

Non-chemical control is the basis of sustainable and effective integrated weed management in highlands. Deeply ploughing with a depth of >20 cm is one of the most effective non-chemical means to manage troublesome weeds, including CAAWs [60,61]. It has been reported that, compared to conventional tillage treatments (to a soil depth of 25–30 cm), experimental plots with shallow ploughing (to a depth of 7–10 cm) and ploughless treatments exhibited 25.5 % and 41.5 % more species of weed seeds in the soil, respectively [60]; and deeply ploughing is also suggest to be effective for managing troublesome CAAWs such as *Xanthium* spp. [62] and *Phalaris minor* [30]. Mulching with plastic film or crop straw is another standard method to manage troublesome weeds in highlands [63,64]. Moreover, mechanical inter- and/or intra-row weeding is an essential means of non-chemical weed control in highlands [65]. Another effective non-chemical weed management strategy is crop rotation between cereal crops and broadleaf crops [66], which significantly increases the effectiveness of chemical control strategies. Panicle-removing control of troublesome weeds is frequently taken by farmers, which could also be used for controlling troublesome CAAWs [82]. Among the above non-chemical methods, deep ploughing has been one of the most critical and practical ways for managing serious CAAWs, such as *Aegilops tauschii*, *Alopecurus myosuroides*, *Lolium multiflorum* and *Avena fatua* in wheat lands, and *Alternanthera* spp. in broadleaf highland crops.

As to chemical control, pre-emergence chemical control is widely used in Chinese wheat lands with several herbicides such as isoproturon, flufenacet, diflufenican, acetochlor, and thiophenesulfuron. As well, post-chemical control is the most important mean of weed management in wheat lands, with various herbicides such as methyldisulfuron-methyl, pinoxaden, clodinafop-propargyl, fenoxaprop-*P*-ethyl, isoproturon, fluroxypyr, halauxifen-methyl, florasulam, and tribenuron [67]. For *Aegilops tauschii* chemical control, isoproturon, flufenacet and methyldisulfuron-methyl could be useful, while methyldisulfuron-methyl-resistant *Aegilops tauschii* have been reported [[69], [70], [71]]. Pyroxasulfone, isoproturon, acetochlor, methyldisulfuron-methyl, clodinafop-propargyl, fenoxaprop-*P*-ethyl, pinoxaden are frequently used for *Alopecurus myosuroides* and *Avena fatua* control, while many populations of *Alopecurus myosuroides* [25,26] and *Avena fatua* [28,29,72] have evolved resistance to methyldisulfuron-methyl, clodinafop-propargyl, fenoxaprop-*P*-ethyl and/or pinoxaden. Isoproturon, flufenacet, diflufenican, methyldisulfuron-methyl and pinoxaden are frequently used for controlling *Lolium multiflorum*, while the efficacies of methyldisulfuron-methyl and pinoxaden against *Lolium multiflorum* are not quite high; as well, *Lolium multiflorum* have evolved resistance to pinoxaden and methyldisulfuron-methyl [15,27]. Diflufenican, acetochlor, thiophenesulfuron, fluroxypyr, halauxifen-methyl, florasulam, and tribenuron are frequently used for controlling broadleaf weeds in wheat lands including *Veronica persica, Geranium carolinianum, Ranunculus muricatus,* and *Cerastium glomeratum* [15,67].

Common pre-emergence herbicides used in Chinese oilseed rape fields include s-metolachlor, acetochlor, pyrometry, clomazone, and oxadiazon; and frequently used post-emergence herbicides including quizalofop-P-ethyl, haloxyfop-P-methyl, fluazifop-P-butyl, sethoxydim, benazolin-ethyl, clopyralid, and picloram [67]. Quizalofop-P-ethyl and haloxyfop-P-methyl are highly effective against CAAWs from the family Poaceae, such as *Aegilops tauschii, Lolium multiflorum, Alopecurus myosuroides, Avena fatua,* while not effective against CAAWs from other families. Sethoxydim also targets Poaceae weeds but is not quite effective against *Lolium multiflorum.* Benazolin-ethyl, clopyralid, and picloram are used for controlling broadleaf weeds [67], while benazolin-ethyl is not quite effective against some broadleaf weeds such as *Veronica persica* and *Geranium carolinianum* [67].

In soybean and peanut lands, frequently used pre-emergence herbicides in China including s-metolachlor, acetochlor, pyrometry, metribuzin, pendimethalin, trifluralin, clomazone and thifensulfuron-methyl, which could be used for controlling most CAAWs occurring in soybean and peanut lands. Frequently used post-emergence herbicides including quizalofop-P-ethyl, haloxyfop-P-methyl, fluazifop-P-butyl, sethoxydim, imazamox, imazethapyr, imazaquin, fomesafen, flumioxazin, oxyfluorfen and acifluorfen [67]. Quizalofop-P-ethyl, haloxyfop-P-methyl, fluazifop-P-butyl, and sethoxydim could be used for controlling CAAWs from family Poaceae, but not for those from other families. Imazamox, imazethapyr, imazaquin, fomesafen, flumioxazin, oxyfluorfen and acifluorfen are frequently used for controlling *Alternanthera philoxeroides*, and *Amaranthus* spp.*, Amaranthus blitum, Abutilon theophrasti,* and *Ageratum conyzoides*.

Various pre-emergence herbicides are frequently used in Chinese cornlands with large areas such as s-metolachlor, acetochlor, pyrometry andhalosulfuron-methyl, which could be used for controlling most CAAWs occurring in cornlands. As to post-emergence herbicides, mesotrione, oxadiazon, atrazine, pyrometry, fluroxypyr, topramezone, fluroxypyr, MCPA, nicosulfuron and thifensulfuron-methyl are frequently used. Fluroxypyr, MCPA, nicosulfuron and thifensulfuron-methyl are frequently used for controlling *Alternanthera philoxeroides*, and *Amaranthus* spp.*, Amaranthus blitum, Abutilon theophrasti,* and *Ageratum conyzoides*. Moreover, herbicide-resistant *Amaranthus retroflexus* have been reported [41,68]. Herbicides suitable for millet and sorghum lands are very limited, particularly for post-emergence chemical control [67].

Integrated weed management strategies could be important in the background of quickly developing herbicide resistance in various weed species including CAAWs. Take *Lolium multiflorum* management in wheat lands in Jiangsu province as an example. The integrative managing strategies include: 1) smashing rice straw during rice harvesting, 2) deeply ploughing before sowing wheat seeds; 3) compacting soil surface after wheat seed sowing with special machinery; 4) applying pre-emergence wheat herbicides before the 1-leaf stage of *Lolium multiflorum* with 132 g a.i. ha^−1^ diflufenican + 132 g a.i. ha^−1^ flufenacet + 132 g a.i. ha^−1^ flurtamone, or 600 g a.i. ha^−1^ isoproturon + 480 g a.i. ha^−1^ acetochlor; 5) apply post-emergence wheat herbicides at the tillering stage of wheat seedlings and the three- to five-leaf stage of *Lolium multiflorum* with 15 g a.i. ha^−1^ mesosulfuron-methyl + 75 g a.i. ha^−1^ pinoxaden + 600 g a.i. ha^−1^ isoproturon; 6) cutting *Lolium multiflorum* panicles before seed maturation, after the bolting stage of wheat; 7) in rice growing season, cleaning irrigation water by intercepting seeds at the water entrance and exit, and removing floating weed seeds with a net during irrigation before rice planting.

### 3.3 Species and practical management strategies of CAAWs in paddy fields

#### 3.3.1 Species of CAAWs in paddy fields

There are six CAAW species in paddy fields in China, including *Paspalum distichum* L., *Panicum dichotomiflorum* Michx., *Alternanthera philoxeroides*, *Ammannia coccinea* Rott., *Bidens tripartita* L.*,* and *Bidens frondosa* L. (Table 1). The occurrence of *Paspalum distichum* at 1005 g m^−2^ caused a rice yield loss of 23.3 % [73], the occurrence of *Alternanthera philoxeroides* at 150 g m^−2^ caused a rice yield loss of 10.2 % [73]; and the occurrence of *Ammannia coccinea* at 32 plants per m^−2^ caused a rice yield loss of 72.3 % [74]. One paddy field growing season is commonly initiated with tillage of the field. Tillage practices, particularly for deep ploughing, effectively destroy the root systems of most weeds, which prevents the vegetative reproduction of most perennial weed species [60,61] and prevents seedling emergence of a great part of soil seed banks [7]. After planting crops on paddy fields, the water layer and alternating wet and dry cause a protective screen against new plant invaders [75,76]. Whereas, the water layer is not effective against *Paspalum distichum*, *Alternanthera philoxeroides*, and *Ammannia coccinea* [14,15]. Most species of rice weeds are native, such as *Echinochloa* spp., *Leptochloa chinensis* (L.) Nees, *Monochoria vaginalis* Burm. f., and *Cyperus difformis* L. [7,77]. Some xerophytic CAAWs may occasionally occur on paddy fields during drought periods or on the edge of paddy fields, such as *Erigeron* spp., *Sonchus asper*, *Bidens* spp., *Solidago canadensis* L., *Amaranthus* spp. and *Ipomoea* spp.

#### 3.3.2 Management strategies against CAAWs in paddy fields

Many weed seeds are distributed among paddy fields by water, which means decreasing the number of weed seeds in irrigating water or on-field water layers could be highly effective in ecologically controlling CAAWs in paddy fields, such as cleaning irrigation water by intercepting seeds at the water entrance and exit and removing floating weed seeds with a net during irrigation before rice planting [78]. Besides, covering it with degradable mulching film to suppress weed occurrence is also done in some organic cropping rice fields [83]. Other non-chemical weed control methods for the highlands mentioned above are also helpful for paddy fields.

Regarding chemical control, *Paspalum distichum* [84,85] and *Panicum dichotomiflorum* [86,87] are from the family Poaceae as rice and cause difficulties for chemical control. Metamifop is reported to be effective for post-emergence control of *Paspalum distichum* and *Panicum dichotomiflorum* [88]. Whereas, metamifop and many rice herbicides targeting grassy weeds could not be applied after the jointing stage of rice, due to the high risks of rice injury and a great decrease in control efficacies against grassy weeds [20]. Thus, Pre-emergence chemical control should be highlighted for controlling *Paspalum distichum* and *Panicum dichotomiflorum.* In eastern China, rice growers frequently control *Paspalum distichum* and *Panicum dichotomiflorum* seedlings and plants in ditches and ridges around fields. *Alternanthera philoxeroides*, *Ammannia coccinea*, *Bidens tripartita,* and *Bidens frondosa* are all broadleaf weed species, which could be controlled by various pre- or post-emergence rice herbicides such as pyrazosulfuron-ethyl, halosulfuron-methyl, ethoxysulfuron, oxyfluorfen, bentazone, MCPA, and fluroxypyr, triclopyr [20]. Therefore, *Alternanthera philoxeroides*, *Ammannia coccinea*, *Bidens tripartita,* and *Bidens frondosa* are relatively easier for chemical control on rice fields [20]. *Alternanthera philoxeroides* is troublesome in many kinds of croplands, while it is sensitive to rice herbicides fluroxypyr and triclopyr. Moreover, *Ammannia coccinea* reproduces a large number of tiny seeds [89], which germinate in homogeneously after rice planting in hot seasons. Thus, *Ammannia coccinea* may still infest rice after applying suitable chemical herbicides, applying pre-emergence herbicides targeting this species multiple times after rice planting could be effective. Conversely, on paddy fields planting broadleaf crops, *Alternanthera philoxeroides*, *Ammannia coccinea*, *Bidens tripartita,* and *Bidens frondosa* could be difficult for post-emergence chemical control for the low selectivity between broadleaf crops and broadleaf weeds [20].

### 3.4 Species and practical management strategies of CAAWs in plantations

Compared with arable lands, plantations frequently host more alien invasive plants with higher occurrence [90]. Crops in plantations are usually perennial, and thus tillage is limited in plantations [7]. Perennial CAAWs may continuously occur in plantations with accumulative seriousness. On the other hand, the occurrence of many alien invasive plant species does not need to be controlled, for the low threats to crops and even benefits for the overall out-puts of plantations [7]. Hence, many alien invasive plant species in plantations are not listed as CAAWs, such as low annuals and biennials (non-climbing plants) with plant heights < 50 cm in most plantations except for nursery gardens. For example, *Trifolium repens* L., an alien invasive plant species in China, is planted in many orchards in China, which provides many benefits such as 1) conserving water and soil by covering the soil surface and increasing soil organic matter, in particular in foothills; 2) biologic fixation of soil nitrogen; 3) acceptable low competitions with fruit trees; 4) being beneficial for harvesting and fruit collection; 5) providing habitats and other benefits for beneficial insects; and 6) suppressing the occurrence of troublesome weeds [27,[90], [91], [92], [93]].

Troublesome weeds in plantations in China mainly include three sorts: 1) tall herbs, shrubs, and woody plants; 2) climbing plants; and 3) plants with spiny stems, leaves or fruits [7,94,95]. Troublesome tall herbaceous CAAW species, mainly including *Solidago canadensis* [96], *Chromolaena odorata* (L.) R. M. King & H. Rob. [12,97,98], *Phytolacca americana* L. [90], *Erigeron* spp. [95], *Bidens* spp. [99], *Parthenium hysterophorus* L., and *Amaranthus retroflexus*. Tall CAAW species including *Solidago canadensis*, *Erigeron canadensis* L., *Erigeron sumatrensis* Retz., *Chromolaena odorata*, *Amaranthus retroflexus*, and *Phytolacca americana*. Climbing CAAWs mainly include *Mikania micrantha* [11,12], *Ipomoea cairica* (L.) Sweet*, Ipomoea triloba, Ipomoea nil,* and *Ipomoea purpurea*. In nursery stocks, *Wedelia trilobata* (L.) Hitchc. could also be troublesome [11,12]. Spiny CAAW species mainly including *Xanthium chinense*, *Bidens* spp., and *Sonchus asper* [99].

Understory management is important for improving the ecological and economic effects of plantations [100,101]. Various plant species are used for understory coverage maintenance in plantations, such as *Paspalum notatum* Flüggé [102], *Pennisetum americanum* (L.) Leeke [102], *Vulpia myuros* (L.) C. C. Gmel. [103] and *Trifolium repens* [93,104]. Some CAAW species are also used for understory management of certain kinds of plantations, such as *Spermacoce alata* Aubl. and *Wedelia trilobata* in orchards in southern China. Clipping is a common non-chemical direct method for weed management in plantations in China [7], which should be highlighted for controlling troublesome climbing CAAWs. For example, *Mikania micrantha* and *Ipomoea* spp. frequently for large population on the crop canopy of plantations, clipping their base stems could be very effective. Considering the quick re-growth of weeds in plantations, clipping is usually not enough to control weed communities, in particular for CAAW species with high ability of vegetative reproduction, such as *Slidago canadensis* [105,106]. Moreover, many plantation owners cover the soil surface with landscape fabric (weed barrier fabric) to prevent weed occurrence [107,108]. Manual removing is a traditional and important method for weed control in plantations.

Chemical control is also one of the most important weed managemental means applied in plantations in China. Glyphosate, glufosinate, and glufosinate-p are the most frequently used non-selective herbicides. Haloxyfop-R-methyl and quizalofop-P-ethyl are frequently used for grassy weed control. Saflufenacil and triclopyr are frequently used for broadleaf control [67]. Nevertheless, applying herbicides might cause serious crop injury in plantations.

## 4 Conclusions

A total of 88 species have been identified as common alien agricultural weeds (CAAWs) in China. Compared to the overall checklist of alien invasive plant species in China, CAAW species exhibit a higher representation of families such as Asteraceae, Poaceae, Amaranthaceae, and Plantaginaceae, which are primarily native to Eurasia and were introduced into China after 1949. Notable species that frequently infest highland areas with summer-ripe crops include *Aegilops tauschii, Alopecurus myosuroides, Lolium multiflorum, Avena fatua, Phalaris minor, Phalaris paradoxa, Veronica persica, Geranium carolinianum, Ranunculus muricatus*, and *Cerastium glomeratum*. In contrast, species such as *Alternanthera philoxeroides, Amaranthus retroflexus, Amaranthus viridis, Amaranthus hybridus, Panicum repens, Paspalum conjugatum,* and *Ageratum conyzoides* are more prevalent in highlands with autumn-ripe crops. Additionally, *Paspalum distichum* and *Bidens tripartita* occasionally infest rice fields. The problematic CAAW species in plantations primarily consist of tall herbs or shrubs, climbing plants, and spiny species. Current management strategies against CAAWs in China mainly rely on chemical control, tillage, soil mulching, and mechanical weeding.

The list of CAAW species is likely to grow in response to the escalating threat of plant invasions in China. Field surveys conducted from 1987 to 1990 in Anhui province recorded 24 alien weed species from 10 families; a subsequent survey approximately 15 years later identified 38 species from 13 families, with a notable doubling in the dominance and richness of alien plant populations [11]. Therefore, it is imperative to establish effective risk assessment systems tailored to agricultural lands, systematically assess weed risks, and identify high-risk CAAW species. Furthermore, elucidating the underlying mechanisms of successful invasions and developing effective, feasible, and sustainable integrated management strategies will be essential in addressing the challenges posed by troublesome CAAWs.

## CRediT authorship contribution statement

**Zeyue Huang:** Writing – review & editing, Writing – original draft, Methodology, Investigation, Formal analysis, Data curation. **Min Lin:** Writing – review & editing, Data curation, Conceptualization. **Guoqi Chen:** Writing – review & editing, Writing – original draft, Project administration, Investigation, Funding acquisition, Conceptualization.

## Data availability statement

Statement Data is contained within the article.

## Funding

This research was funded by a project funded by Jiangsu Key R&D Plan (BE2022338) and the 10.13039/501100012246Priority Academic Program Development of Jiangsu Higher Education Institutions (PAPD).

## Declaration of competing interest

The authors declare that they have no known competing financial interests or personal relationships that could have appeared to influence the work reported in this paper.Guoqi Chen reports financial support was provided by 10.13039/501100007062Yangzhou University. Guoqi Chen reports a relationship with Yangzhou University that includes: employment. If there are other authors, they declare that they have no known competing financial interests or personal relationships that could have appeared to influence the work reported in this paper.
